# The Adaptation for Conservation Targets (ACT) Framework: A Tool for Incorporating Climate Change into Natural Resource Management

**DOI:** 10.1007/s00267-012-9893-7

**Published:** 2012-07-07

**Authors:** Molly S. Cross, Erika S. Zavaleta, Dominique Bachelet, Marjorie L. Brooks, Carolyn A. F. Enquist, Erica Fleishman, Lisa J. Graumlich, Craig R. Groves, Lee Hannah, Lara Hansen, Greg Hayward, Marni Koopman, Joshua J. Lawler, Jay Malcolm, John Nordgren, Brian Petersen, Erika L. Rowland, Daniel Scott, Sarah L. Shafer, M. Rebecca Shaw, Gary M. Tabor

**Affiliations:** 1 Wildlife Conservation Society, 301 N. Willson Avenue, Bozeman, MT 59715 USA; 2 Environmental Studies Department, University of California, Santa Cruz, CA USA; 3 Conservation Biology Institute, Corvallis, OR USA; 4 Department of Zoology, Southern Illinois University, Carbondale, IL USA; 5 The Wildlife Society, Bethesda, MD USA; 6 USA National Phenology Network, Tucson, AZ USA; 7 Bren School of Environmental Science & Management, University of California, Santa Barbara, CA USA; 8 John Muir Institute of the Environment, University of California, Davis, CA USA; 9 College of the Environment, University of Washington, Seattle, WA USA; 10 The Nature Conservancy, Bozeman, MT USA; 11 Center for Applied Biodiversity Science (CABS), Conservation International, Arlington, VA USA; 12 EcoAdapt, Bainbridge Island, WA USA; 13 Rocky Mountain Regional Office, U.S. Forest Service, 740 Simms Street, Golden, CO USA; 14 Geos Institute, Ashland, OR USA; 15 School of Environmental and Forest Sciences, University of Washington, Box 352100, Seattle, WA USA; 16 Faculty of Forestry, University of Toronto, Toronto, ON Canada; 17 Kresge Foundation, Troy, MI USA; 18 Michigan State University, Kellogg Biological Station, East Lansing, MI USA; 19 Wildlife Conservation Society, Tucson, AZ USA; 20 University of Waterloo, Waterloo, ON USA; 21 U.S. Geological Survey, Corvallis, OR USA; 22 Environmental Defense Fund, San Francisco, CA USA; 23 Center for Large Landscape Conservation, Bozeman, MT USA

**Keywords:** Adaptation, Climate change, Conservation, Decision-making, Management, Natural resources

## Abstract

As natural resource management agencies and conservation organizations seek guidance on responding to climate change, myriad potential actions and strategies have been proposed for increasing the long-term viability of some attributes of natural systems. Managers need practical tools for selecting among these actions and strategies to develop a tailored management approach for specific targets at a given location. We developed and present one such tool, the participatory Adaptation for Conservation Targets (ACT) framework, which considers the effects of climate change in the development of management actions for particular species, ecosystems and ecological functions. Our framework is based on the premise that effective adaptation of management to climate change can rely on local knowledge of an ecosystem and does not necessarily require detailed projections of climate change or its effects. We illustrate the ACT framework by applying it to an ecological function in the Greater Yellowstone Ecosystem (Montana, Wyoming, and Idaho, USA)—water flows in the upper Yellowstone River. We suggest that the ACT framework is a practical tool for initiating adaptation planning, and for generating and communicating specific management interventions given an increasingly altered, yet uncertain, climate.

## Introduction

Scientists, managers, and decision makers worldwide have advocated for the development of innovative approaches to minimize the effects of climate change on species, ecosystems, and ecological functions (e.g., Mitchell and others 2007; US-GAO 2007; Campbell 2008). General principles for maintaining the viability of species and ecosystems over the long term include increasing the size and number of reserves, increasing connectivity of species’ habitats, reducing stressors other than climate change (e.g., pollution, habitat fragmentation), and applying adaptive management (Scott and Lemieux 2005; Mawdsley and others 2009; West and others 2009; Hansen and others 2010). These principles largely lack the specificity needed to direct on-the-ground implementation (Heller and Zavaleta 2009). There is therefore a need for practical planning approaches that help transform general recommendations for adaptation of human actions into site- and target-specific strategies for action (Enquist and others 2009).

Broad instructions and guidance on adaptation planning have been put forth by state and federal natural resource management agencies in the United States (e.g., AFWA 2009; CEQ 2011; Peterson and others 2011). These resources outline general steps such as assessing potential effects of climate change, and developing, prioritizing and implementing adaptation actions; but do not include specific methods. Other guides provide methods for assessing climate change vulnerability (e.g., Glick and others 2011), but do not elaborate on how to use that information to identify adaptation options.

More detailed adaptation-planning methods are beginning to emerge (e.g., Willows and Connell 2003; Ogden and Innes 2009; NOAA 2010; Halofsky and others 2011; Poiani and others 2011; Weeks and others 2011; Groves and others 2012), none of which can meet all decision-making needs of natural resource managers in all situations. For example, the U.S. Forest Service convened a science-management partnership over the course of 1.5 years to assess climate change vulnerability and adaptation options for hydrology, roads, fish, wildlife, and vegetation (Halofsky and others 2011), but only addressed management of federal lands and provided limited guidance on incorporating uncertainty into the planning process. The U.S. National Park Service (NPS) has developed a scenario planning approach that explicitly addresses uncertainties in climate change and other key drivers of management decisions (Weeks and others 2011). To date, the NPS scenario planning efforts have focused on general adaptation strategies for a breadth of natural and cultural resources in National Parks, rather than on targeted management options for specific resources. The Nature Conservancy (TNC) has developed steps to address climate change in conservation strategies for focal species and ecosystems (Poiani and others 2011), but applying the steps requires familiarity with TNC’s Conservation Action Planning method (TNC 2009) and an existing conservation plan derived with that method.

As a complement to these and other tools that might support adaptation planning, we present the Adaptation for Conservation Targets (ACT) framework. Its novel contribution is derivation of place-based adaptation actions for particular species, ecosystems, and ecological functions through a simple process that encourages participation of multiple public and private jurisdictions. The ACT framework can be used where any degree of formal conservation planning has already occurred, and considers multiple future scenarios to address uncertainty. It can function as a stand-alone planning process, or it can be used to integrate climate change into existing decision-making and strategic planning processes.

**Fig. 1:**
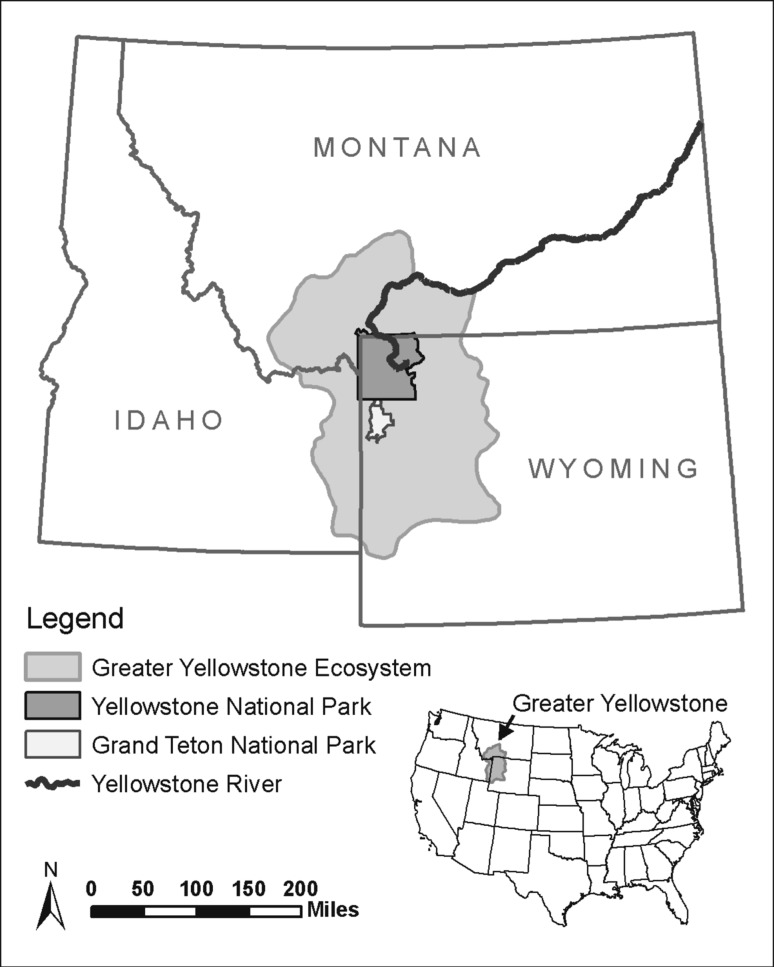
Map showing the Greater Yellowstone Ecosystem and the Yellowstone River in Montana, Wyoming, and Idaho, USA. (Map created by A. Toivola, Wildlife Conservation Society)

In developing the ACT framework, we drew on familiar decision-support tools to increase the likelihood and ease of adoption. These tools include structured decision-making (e.g., Ohlson and others 2005), adaptive management (e.g., Conroy and others 2011), and the Open Standards for the Practice of Conservation (CMP 2007). The ACT framework is also informed by approaches for adapting to climate change in other sectors including tourism (Simpson and others 2008), water management (e.g., Johnson and Weaver 2009), forestry (e.g., Spittlehouse and Stewart 2003), economic development (e.g., USAID 2007), and community sustainability (e.g., Snover and others 2007). By combining elements of familiar tools and approaches, ACT aims to accelerate place-based adaptation planning for natural resources and allow for the immediate integration of projected effects of climate change and associated uncertainties into management decisions. We describe the ACT steps and illustrate its application to a conservation target in the Greater Yellowstone Ecosystem (Montana, Wyoming, and Idaho, USA) (Fig. 1).

## Adaptation for Conservation Targets (ACT)

**Fig. 2:**
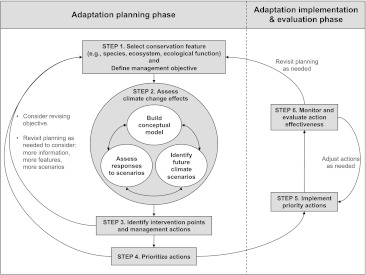
The Adaptation for Conservation Targets (ACT) framework for natural resource management planning in light of climate change. Steps 1–4 represent the ACT planning phase (the focus of this paper); Steps 5–6 represent the implementation and evaluation phase

The ACT framework is designed to motivate collaborative, scientifically defensible planning and decision-making for specific landscapes or seascapes by a multidisciplinary group of practitioners. Participants with extensive, local expertise and a mandate to make management decisions are essential for the process to be effective. The framework is a simple yet structured approach that builds familiar elements of natural resource planning (e.g., local knowledge, conceptual modeling, and adaptive management) into a process tailored for addressing climate change (Fig. 2):

Step 1. Identify the feature targeted for conservation (e.g., species, ecosystem or ecological function) and specify a management objective for that feature;
Step 2. Assess the potential effects of plausible future climate scenarios on that feature:
  • Build a conceptual model that illustrates the climatic, ecological, social, and economic drivers affecting the feature;
Build a conceptual model that illustrates the climatic, ecological, social, and economic drivers affecting the feature;
Develop a suite of plausible climate change scenarios;
Examine how the feature and its non-climatic drivers may respond to each scenario.
Step 3. Identify management actions to achieve the stated objective under each scenario;
Step 4. Prioritize management actions;
Step 5. Implement priority actions; and
Step 6. Monitor action effectiveness and progress toward objectives; adjust ineffective actions or revisit planning as needed.

Following the basic approach of adaptive management cycles (e.g., Williams and others 2009), ACT steps can be repeated to monitor and project changes in management and social priorities, climate trajectories, and ecological responses. Information needs identified throughout the process can yield a priority research agenda, but need not prevent progress towards implementing management actions. Although the framework includes both a planning phase and an implementation and evaluation phase (Fig. 2), we focus here on the planning phase (Steps 1–4). We chose the Greater Yellowstone Ecosystem (GYE) for a rapid pilot test of the planning phase. Examples from this pilot are intended to illustrate the planning steps rather than prescribe management actions.

## Planning Steps

### Step 1: Identify Conservation Feature and Management Objective

The first step in the ACT framework is to select a feature (i.e., species, ecosystem, ecological function) of interest. The selection could depend on existing priorities, a management mandate, or a feature’s likely response to changing climate. Initially focusing on a single feature (or a finite set of related features), rather than simultaneously considering all of the species or ecosystems within a planning area, increases the feasibility of planning. It also allows managers to translate abstract concepts of how climate and ecosystems may change into a more concrete understanding. As time and resources allow, additional features can be addressed incrementally to provide a more complex representation of an area or ecosystem of interest.

For the GYE pilot, we chose to focus on upper Yellowstone River water flows (Fig. 1). Although many management objectives could be linked to the ecological function of water flows, we specified an objective of sustaining flow conditions suitable for native Yellowstone cutthroat trout (*Oncorhynchus clarkii bouvieri*), a management focus and species of concern for several state and federal agencies in the region. These conditions include peak spring flows that support spawning and limit interactions with non-native rainbow trout (*Oncorhynchus mykiss*) and late summer flows that maintain water temperatures within the optimum range for the species (4–15 °C) (Gresswell 2009).

### Step 2: Assess Effects of Plausible Future Climate Scenarios

The ACT framework emphasizes reliance on local ecological knowledge through the use of graphical conceptual models and expert opinion-based assessments of climate change effects and management options, supplemented by scientific literature. This part of the planning process is nonlinear because the processes of identifying key drivers, developing plausible scenarios, and synthesizing information on potential ecological responses inform each other.

#### Build Conceptual Model

**Fig. 3:**
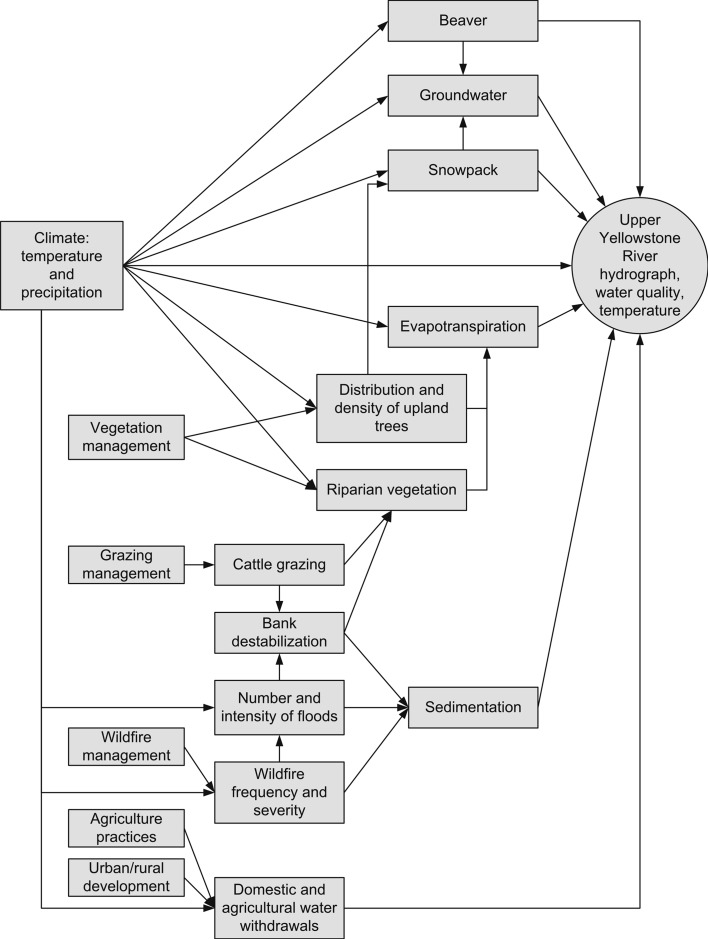
Conceptual model illustrating how climate and other drivers may influence water flows in the upper Yellowstone River

A graphical conceptual model identifies participants’ assumptions about the feature’s current and potential future ecological, physical, climatic, social, and economic drivers (Margoluis and others 2009) (see Fig. 3). Participation by relevant experts helps ensure that the most important drivers are identified. Guidance for the transparent construction of conceptual models (e.g., Foundations of Success 2009) can be applied during this step. Assessments that elucidate the specific factors that affect a species’ or ecosystem’s potential response to climate change (e.g., Glick and others 2011; Rowland and others 2011) can identify which climate-associated drivers should be incorporated in the conceptual model. By including natural and human-related drivers other than climate, the conceptual model highlights the interaction of climate change with other stressors.

#### Develop Plausible Climate Scenarios

One challenge to integrating climate change into conservation planning is that there are many projections of future climate conditions produced by different climate models, scenarios of greenhouse gas emissions, and methods for increasing the resolution of projections. There are also fundamental and irreducible uncertainties in projecting future climate resulting from different assumptions that underlie different climate models, incomplete knowledge, and the complexity of interactions between key system drivers (Coreau and others 2009; Dessai and others 2009). This argues for consideration of multiple plausible climate scenarios in adaptation planning (IPCC-TGCIA 1999; Peterson and others 2003).

Climate scenarios can range from relatively qualitative narratives about changes in climate (e.g., “warmer with increased precipitation” vs. “warmer with decreased precipitation”) to more quantitative and spatially explicit simulations based on global or regional climate models. Scenarios can incorporate average climate trends and changes in the frequency and intensity of extreme climate events (e.g. droughts, floods), allowing one to address both incremental and abrupt shifts in ecosystem structure or function. Alternate climate scenarios often hinge on uncertainties in climate projections (e.g., discrepancies across climate models in projected changes in magnitude, direction or seasonal timing of precipitation changes). Although it is difficult to plan for conditions and species assemblages for which there are no current analogs (Williams and others 2007), the use of scenarios offers an opportunity to consider future conditions that go beyond the constraints of explicit model projections (Peterson and others 2003).

If warranted, climate scenarios can be integrated with scenarios of change in other stressors or system drivers (e.g., Mahmoud and others 2009). In situations where the effects of climate change on the feature (see below) are uncertain, it is also possible to embed plausible physical, biological and ecological responses into the climate change scenarios. For example, rather than solely focusing on scenarios of changes in amount of precipitation, planning could consider the effects of alternate scenarios of vegetation response (e.g., species composition of trees in a forest remains the same, species composition shifts, or trees are replaced by grasses).

Although high-resolution climate change information is often desired, there are caveats associated with deriving those data (e.g., see Wiens and Bachelet 2010). With the ACT framework, planning can begin with climate information that is most readily available, even relatively qualitative or non-spatial scenarios of climate change. These scenarios are useful for identifying potential management options, and initial results can inform whether more detailed or spatially explicit climate information is needed to improve understanding of the interactions between climate and other stressors or make particular management decisions. For the GYE pilot, we relied on the opinion of experts on climate model projections for the region to outline plausible scenarios. We specified an initial climate scenario consistent with most projections for western North America in 2020–2030 (IPCC 2007a): increased temperature, reduced snowpack, and reduced precipitation. Because climate models project different directions and magnitudes of precipitation changes for the region (IPCC 2007a), we also considered whether an alternate scenario of increased temperature but moderately increased precipitation would result in different effects on river flows and management recommendations.

#### Examine Responses of Feature to Scenarios

As with the development of future climate scenarios, it may be sufficient to estimate qualitatively the direction and magnitude of direct and indirect effects of the climate scenarios on the feature. A quantitative ecological model that includes all or even most of the drivers identified in the conceptual model may not exist, or may not have been run using the selected climate scenarios as inputs. The ACT framework therefore relies more heavily on expert-driven syntheses of both regional and local ecological knowledge and the experts’ own or related climate change research to assess potential effects. The collective knowledge of participating experts integrates across a range of relevant information generated from analyses of observations, experiments, paleoecological studies, and predictive models.

If targeted quantitative analyses (e.g., predictive models) of the potential effects of climate change on the feature are available, that information can be incorporated into the ACT approach (see *Discussion*). Initial application of the ACT steps on the basis of relatively qualitative inputs can provide a foundation for identifying some actions, while pinpointing instances where it may be justified to generate more quantitative information to inform specific management decisions. Whether climate change effects are assessed using quantitative or qualitative methods, it is important to consider the potential for non-linearity, boundary conditions, thresholds and feedbacks.

For the GYE pilot, we qualitatively synthesized our collective knowledge of available research to assess potential climate change effects. If regional climate becomes warmer and drier, we expect snowpack in the GYE to decrease, spring flows and flood pulses on the Yellowstone River to peak earlier, summer baseflows to decrease, and late summer water temperatures to increase (Stewart and others 2004; Mote and others 2005; Knowles and others 2006). Whereas long periods of low flow have occurred on the Yellowstone River over the past 300 years (Graumlich and others 2003), climate change will superimpose a long-term warming trend on natural flow variability (Milly and others 2008). This would decrease summer baseflow relative to the past century through increased evaporation and decreased snowpack, even if precipitation increases moderately (Stewart and others 2004). Therefore, we expect scenarios of both increased and decreased precipitation to drive water flow in a direction that will negatively affect Yellowstone cutthroat trout.

### Step 3. Identify Management Actions

**Fig. 4:**
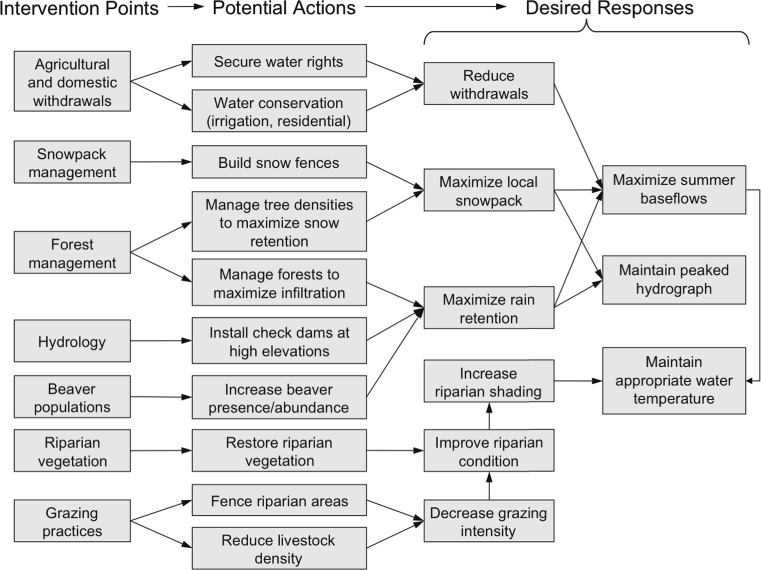
Example results chains for management options and intermediate effects to maintain Yellowstone River flows suitable for Yellowstone cutthroat trout as the climate becomes warmer and drier. Management options are then examined to determine tradeoffs and set priorities (Step 4, Fig. 2)

The conceptual model can be used to identify intervention points—those elements of the system that can be manipulated. For the upper Yellowstone River, intervention points include urban and agricultural withdrawals, hydrology, beaver (*Castor canadensis*) presence and activity, snowpack, cattle grazing, riparian vegetation, forest composition and structure, agricultural practices, and wildfire (Fig. 3). We use results chains (sensu Margoluis and others 2009) to illustrate our knowledge or hypotheses of how multiple actions at those intervention points may help maximize summer baseflows and maintain a peaked hydrograph and water temperatures consistent with occupancy of Yellowstone cutthroat trout as climate changes (Fig. 4). Results chains draw explicit links between potential management actions and assumptions about the intermediate and ultimate effects of those actions, thereby making the decision-making process more transparent. If no actions are capable of achieving the stated objective under most or any of the climate change scenarios considered, users may be required to reevaluate and revise their objective or consider redirecting their resources to other features (Fig. 2).

One goal of this step is to pinpoint which general adaptation principles are most applicable to the feature, and translate those principles into as many concrete management actions as possible, without regard to regulatory, social or economic constraints (because those can be addressed in the prioritization step below). For example, we rendered the general adaptation principle of reducing non-climate stressors into specific actions to manage Yellowstone River flows such as decreasing intensity of cattle grazing in riparian areas and reducing the amount of water withdrawn for agricultural and residential uses. Another goal of this step is to identify actions that are likely to be effective under most or all scenarios. Because we expect flow conditions for Yellowstone cutthroat trout to be negatively affected whether precipitation increases moderately or decreases, the actions we identified (Fig. 4) are applicable under both climate scenarios considered.

### Step 4: Prioritize Management Actions

Once potential management actions are identified, it is necessary to prioritize the actions by their relative feasibility and desirability. Prioritization criteria might include potential for utility across alternate climate scenarios; relative contribution to achieving a particular objective; economic, social and political feasibility; potential for positive synergies or negative unintended consequences; reversibility; and departure from current management practice (e.g., USAID 2007). Prioritization criteria can be applied subjectively on the basis of the opinion of experts involved in planning, or quantitatively when appropriate information is available. If participants are committed to making joint decisions, then the entire group may undertake a collaborative prioritization effort. If not, participants may choose to separately prioritize adaptation options on the basis of relevance to their organization’s goals and objectives.

For the GYE pilot, we qualitatively assessed several costs and benefits associated with three potential actions for maintaining upper Yellowstone River flows: installing snow fences, building check dams, and increasing the presence and abundance of beaver (Table 1). One can then compare tradeoffs across actions and across prioritization criteria to determine which actions to implement. For example, if one is most concerned about economic or social feasibility, or the potential for unintended consequences, then snow fences might be seen as the most viable option. If magnitude of contribution to achieving management goals as climate changes is a priority, then beaver- or man-made dams might need to be considered because they have a more direct effect on streamflows, despite some of the undesirable effects of those actions. As with any participatory planning process, which actions are selected will depend on who is participating in the process and who has decision-making authority.

**Table 1:** Costs and benefits associated with three potential actions for managing Yellowstone River water flows given a warmer and drier climate

		Management actions		
		Install snow fences	Construct check dams	Increase beaver presence/abundance
Prioritization criteria	Contribution to achieving management objective	Relatively indirect positive effect on base flow	Relatively direct positive effect on flows in the upper river drainage	Relatively direct positive effect on flows in the upper river drainage
	*Feasibility*:			
	Economic	Inexpensive	Moderately expensive, but not prohibitive	Relatively inexpensive
	Regulatory	Might not be allowed in portions of watershed within Yellowstone National Park	Might not be allowed in portions of watershed within Yellowstone National Park	Not prohibited in Yellowstone National Park
	Social	Little conflict with downstream users	May conflict with downstream users	Potential conflict with private landowners; will vary among locations
	Potential unintended consequences	No effects on fish passage	Can increase siltation or prevent fish passage	Populations might need to be heavily managed, might prevent fish passage, might migrate into other streams
	Synergies with other management objectives	Delays timing of spring peak flow while increasing summer base flows	–	May improve status of riparian systems
	Potential for removal or modification	High	Becomes more difficult over time, has long-term effects	Difficult, especially over time; has long-term effects
	Consistency with current management practice	Existing tool for other purposes	Existing tool	Existing tool
	Robustness to uncertainty in future climate projections*(e.g., increased rather than decreased precipitation)*	Would still increase base flow	Would still increase base flow, potentially higher risk of blowouts during high flow events	Would still increase base flow, potentially higher risk of blowouts during high flow events

One can compare tradeoffs across actions and prioritization criteria to determine which actions to implement

The ACT framework is best suited for identifying management actions that would be defensible under all or most future climate scenarios and current conditions, or actions that bring high benefits under multiple scenarios of climate change or for multiple objectives, with relatively low costs or risks (Willows and Connell 2003; Smith and others 2009). For example, all of the actions presented in Table 1 may be considered robust across the two climate scenarios we considered because they will continue to increase baseflows if precipitation increases or decreases, although beaver and check dams may become more susceptible to blowouts during high flow events if precipitation increases. To make decisions about actions that are recommended for only a subset of future scenarios, it may be necessary to incorporate other approaches, beyond scenario planning, into this prioritization step (see “Discussion” section).

## Discussion

The ACT framework is intended to address the need for practical adaptation planning approaches that are time- and cost-effective, can be incorporated into existing planning processes, and do not require extensive training. For the upper Yellowstone River pilot, we crafted a conceptual model, evaluated the effects of two plausible climate change scenarios, and identified actions that made sense under both scenarios in less than two days and using relatively limited information about future climate conditions. In this way, the ACT steps help one initiate adaptation planning and move beyond the paralysis that one may feel when dealing with climate change. Because ACT draws heavily on existing local expertise and readily available research, planning can be initiated without extensive investment in new modeling or research. The flexibility and relative simplicity of the ACT steps allows for easier integration into standard planning processes (IPCC 2007b). Many professionals have experience with conceptual models, participatory planning processes, threats assessments, and methods for making decisions under uncertainty.

There are some limitations to the ACT approach. Use of relatively qualitative expert-based information can streamline the planning process. However, there will be instances where more quantitative input to decision-making will be necessary or desired. For example, although our relatively qualitative assessment of potential responses of river flows to environmental change in the GYE pilot allowed us to identify a variety of relevant adaptation actions, it did not reveal whether water temperature thresholds for Yellowstone cutthroat trout might be exceeded under either future climate scenario. This information is necessary to determining whether there are actions managers can take to allow the long-term persistence of Yellowstone cutthroat trout.

One way to address this limitation could be to consider whether the actions we propose are likely to remain effective even if water temperature thresholds are crossed. In the case of the Yellowstone River, many of the actions we suggest for reducing the negative effects of climate change on water flows for the Yellowstone cutthroat trout (Fig. 4) would also likely contribute to the creation of aquatic habitat for warmer-water fish species, such as smallmouth bass (*Micropterus dolomieu*) that already inhabit the lower Yellowstone River and that may replace cutthroat trout as temperatures increase. Alternatively, it may be possible to integrate more quantitative analyses (e.g., spatially explicit projections of air and water temperatures) into the ACT steps. For example, results from climate envelope models, ecosystem models, process models, or other types of models can be directly incorporated into the assessment of climate change responses (Step 2). Tools such as Bayesian belief networks (e.g., Marcot and others 2006) could also be used to quantify the relationships depicted in conceptual models developed by participating experts (Step 2).

Although the ACT framework does highlight relatively robust actions that are recommended across multiple plausible futures, it does not directly help one prioritize and decide whether to take actions that are only recommended under a subset of future scenarios. Prioritizing among actions can also be challenging in situations where the ACT framework is used to facilitate planning for a group of participants that have differing or conflicting missions. The ACT framework may therefore be most useful as a tool to initiate dialogue on adaptation, identify a number of potential adaptation options and implement those actions in the near-term that are recommended under all or most future scenarios and management goals, and monitor for the conditions that might trigger other actions as the future unfolds. It will likely need to be embedded within other decision-making frameworks—such as risk assessment and management (e.g., Willows and Connell 2003) or structured decision-making (Martin and others 2011)—to help managers select among adaptation options that differ across future scenarios or goals.

### Testing and Refining the ACT Framework

Although the GYE pilot illustrated the ACT planning steps and suggests that they provide a useful starting point for adaptation planning, it was not intended to develop prescriptive management recommendations or test the approach in a participatory setting. Initial efforts to more thoroughly test and refine the ACT framework have engaged science experts and managers in planning for a range of conservation features in the GYE, the transboundary USA-Canada Rocky Mountains, New Mexico, Colorado, Arizona, Utah, New York, and the Great Plains Landscape Conservation Cooperative region. These efforts have involved diverse participants, including federal, state, provincial, and tribal natural resource managers, university and governmental scientists, and members of non-governmental organizations. Our experiences suggest that the approach has merit for initiating multi-jurisdictional adaptation planning for natural resources. For example, the Southwest Climate Change Initiative (SWCCI) hosted two-day workshops in New Mexico, Colorado, Arizona and Utah in which scientists and managers from multiple agencies and organizations were led through the ACT planning steps (Cross and others, accepted). Participants at these workshops enumerated potential adaptation actions for two future climate scenarios. At the end of the SWCCI workshop in Gunnison, Colorado, 88% of participants who responded to an exit survey (*n* = 34) indicated that they felt the ACT framework was ‘mostly’ or ‘absolutely’ useful for developing climate adaptation strategies (Cross and others, accepted).

## Conclusion

The ACT framework offers an efficient and structured process for translating broad adaptation principles (e.g., minimize non-climate stressors, monitor to detect changes, intensively manage populations, or increase the size and number of reserves) into actionable management strategies. It does not necessarily require complex modeling, certainty in climate projections, or extended planning time. It does, however, require local knowledge of the system of interest, management expertise, and a basic understanding of readily available climate projections and their limitations. It is transferable among ecological systems and organizations, and its steps can be integrated with existing decision-making processes and other planning tools. It will not provide a single solution for addressing climate change impacts in a system, but it can highlight options that can be explored, evaluated, and tested to inform subsequent management actions. We recognize that given the uncertainties associated with climate and ecological response modeling, it will be particularly important to monitor the effectiveness of management actions and adjust actions accordingly (Step 6, Fig. 2) (Lawler and others 2010; Conroy and others 2011).

Increasingly sophisticated climate science will not increase the probability of achieving management objectives if institutions and regulations constrain the implementation of adaptation strategies (Hannah and others 2002; Scott and others 2002; Moser and Ekstrom 2010). Social and political changes that foster cooperation within and across jurisdictions, and increase the social capacity for adaptation, will ultimately be necessary. Consequently, a critical first step is to convene diverse partners to identify proactive adaptation strategies within existing constraints. The ACT framework has the potential to overcome some of the significant, real barriers that continue to prevent practitioners from moving toward adaptation of their actions to climate change.
